# Indoor visual SLAM dataset with various acquisition modalities

**DOI:** 10.1016/j.dib.2021.107496

**Published:** 2021-10-19

**Authors:** Imad El Bouazzaoui, Sergio Rodriguez, Bastien Vincke, Abdelhafid El Ouardi

**Affiliations:** SATIE - CNRS UMR 8029, Paris-Saclay University, France

**Keywords:** Depth map, Indoor localization, RGB-D cameras, Robotics

## Abstract

The indoor Visual Simultaneous Localization And Mapping (V-SLAM) dataset with various acquisition modalities has been created to evaluate the impact of acquisition modalities on the Visual SLAM algorithm’s accuracy. The dataset contains different sequences acquired with different modalities, including RGB, IR, and depth images in passive stereo and active stereo modes. Each sequence is associated with a reference trajectory constructed with an Structure From Motion (SFM) and Multi View Stereo (MVS) library for comparison. Data were collected using an intrinsically calibrated Intel RealSense D435i camera. The RGB/IR and depth data are spatially aligned, and the stereo images are rectified. The dataset includes various areas, some with low brightness, with changes in brightness, wide, narrow and texture.

## Specifications Table

**Table:** 

Subject	Computer Science
Specific subject area	Computer Vision and Pattern Recognition
Type of data	Image
	Reference trajectory : text file
	Timestamps files : text file
	Intrinsic parameters files (ORB-SLAM2 parameters format [1]) : yaml file
How data were acquired	Images were acquired using a Intel RealSense D435i camera connected to a machine equipped with an Intel Celeron N4100 Quad-Core CPU, 8G RAM and 512GB SSD memory, runing Ubuntu 18.04 and Realsense viewer app for Digiteo_seq1 and Digiteo_seq2. Digiteo_seq3 was acquired using a laptop equipped with AMD® Ryzen 9 4900hs with 23GB RAM and 1TB SSD runing Ubuntu 20.04 and ROS Noetic. The experiment was carried out using different acquisition modes. The sensor has been intrinsically calibrated in advance using Intel® RealSense™D400 Series Dynamic Calibration Tool to obtain a spatial alignment between the RGB/IR frames and the depth frames, and to have already rectified stereo frames.
Data format	Analysed
Parameters for data collection	Indoor acquisitions were performed, taking into account different scene light conditions, various scene scale, textured, and low-textured environments.
Description of data collection	Indoor images were acquired using various acquisition modes, including IR stereo, RGB-D active stereo (IR projector on), RGB-D passive stereo (IR projector off), and IR-D passive stereo. IR-D was not recorded in active stereo because the IR patterns interfere with feature extraction, causing spurious detections. Images were recorded with a resolution of 1280x720 pixels and a frame rate of 30 FPS. RGB images have been converted to grayscale to reduce data size. The difference between the RGB images and the IR is the field of view and exposure modes. It is a rolling shutter for the RGB camera, while for the IR, it is a global shutter.
Data source location	Institution: SATIE Laboratory - Paris-Saclay University
	City/Town/Region: Gif-sur-Yvette
	Country: France
	Latitude: 48.71264, Longitude: 2.16825
Data accessibility	Repository name: Mendeley Data
	Data identification number:
	10.17632/7swv73drgr.3
	10.17632/tb9g7th9yz.2
	10.17632/c2gtvyxyt7.2
	10.17632/2n7j5pg2xj.2
	10.17632/kpps3854xm.2
	10.17632/5xmzkgcgg7.2
	Direct URL to data:
	https://doi.org/10.17632/7swv73drgr.3
	https://doi.org/10.17632/tb9g7th9yz.2
	https://doi.org/10.17632/c2gtvyxyt7.2
	https://doi.org/10.17632/2n7j5pg2xj.2
	https://doi.org/10.17632/kpps3854xm.2
	https://doi.org/10.17632/5xmzkgcgg7.2
Related research article	I. El Bouazzaoui, S. Rodriguez and A. El Ouardi, “Enhancing RGB-D SLAM Performances Considering Sensor Specifications for Indoor Localization,” in IEEE Sensors Journal, doi: 10.1109/JSEN.2021.3073676.

## Value of the Data

• This dataset contains three sequences. Each sequence contains two to four acquisition modalities in the same environment, allowing to visualize the sensor’s impact on the localization accuracy of SLAM algorithms.
• The dataset is relevant to the computer vision and robotics field, particularly for autonomous robot applications involved in localization in an indoor environment.
• The provided dataset can be used to evaluate visual SLAM algorithms or visual odometry algorithms, with different input types such as monocular, stereo, or RGB-D.
• The data is temporally and spatially aligned and ready to be used without further pre-processing.

## 1 Data Description

**Fig. 1:**
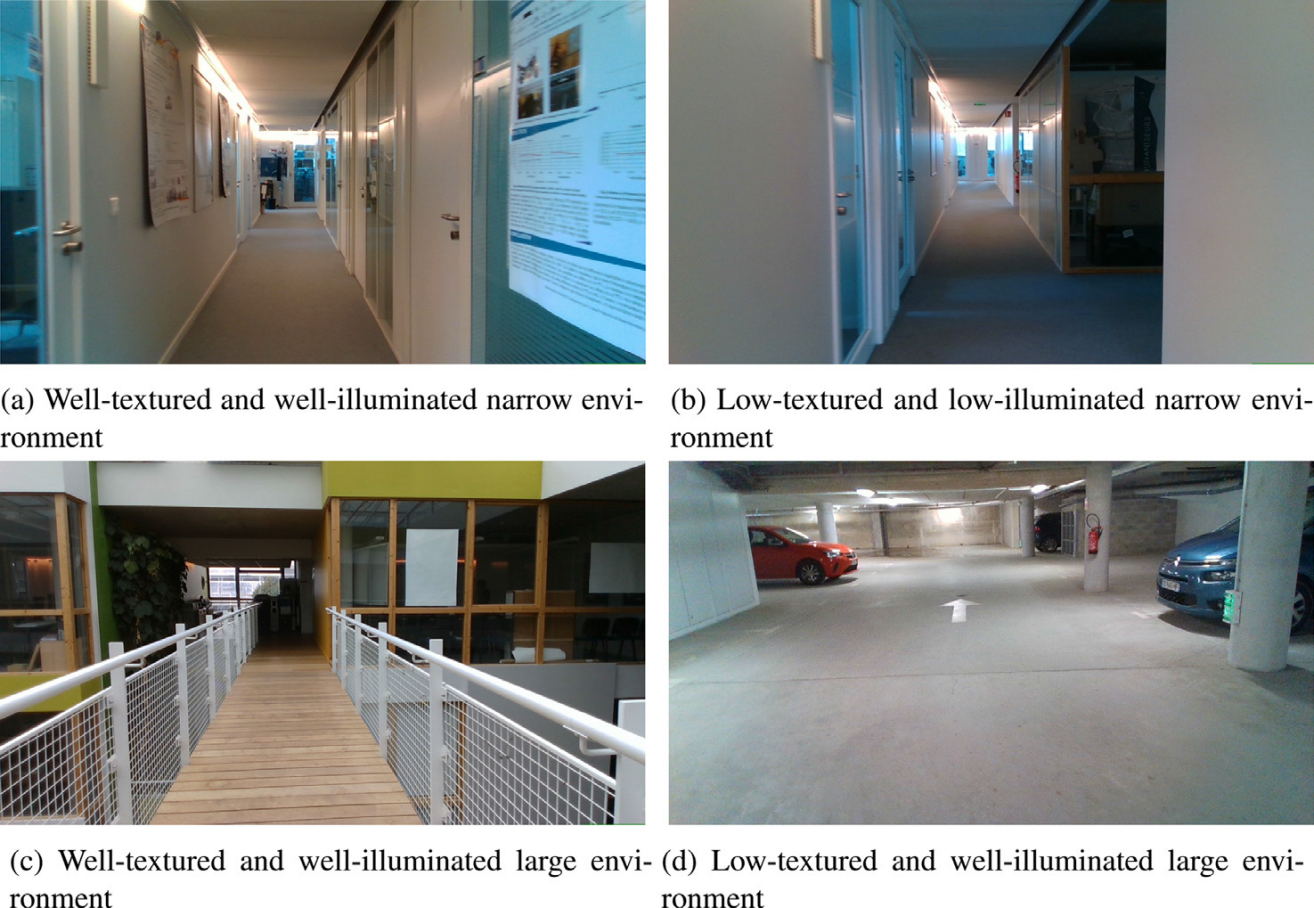
Different images from Digiteo_seq1, Digiteo_seq2 and Digiteo_seq3.

The simultaneous localization and mapping problem known as SLAM is considered one of the pillars of autonomy in robotics and autonomous vehicles, besides other applications that use it. This problem has been under hard work for over a decade. Several solutions have been proposed using various algorithms with a variety of sensors, such as [1], [2], [3]. With the development of SLAM algorithms, several datasets have been made available to researchers to evaluate their algorithms, particularly visual SLAM. Often these datasets are intended for evaluation of the algorithm with a single acquisition modality, such as [4], [5]. As a result, there are no different acquisition modalities for the same sequence, allowing us to compare the sensor’s impact on the localization accuracy. In this work, we propose a dataset consisting of three sequences with different acquisition modalities. The dataset was the subject of a study done in [6]. The dataset includes three static sequences in two different environments. Two sequences are recorded in the laboratory’s corridors: Digiteo_seq1 and Digiteo_seq2, as shown in the Figs. 1a–1c. Furthermore, one sequence is recorded in the basement parking of the laboratory: Digiteo_seq3, as illustrated in Fig. 1d.

**Fig. 2:**
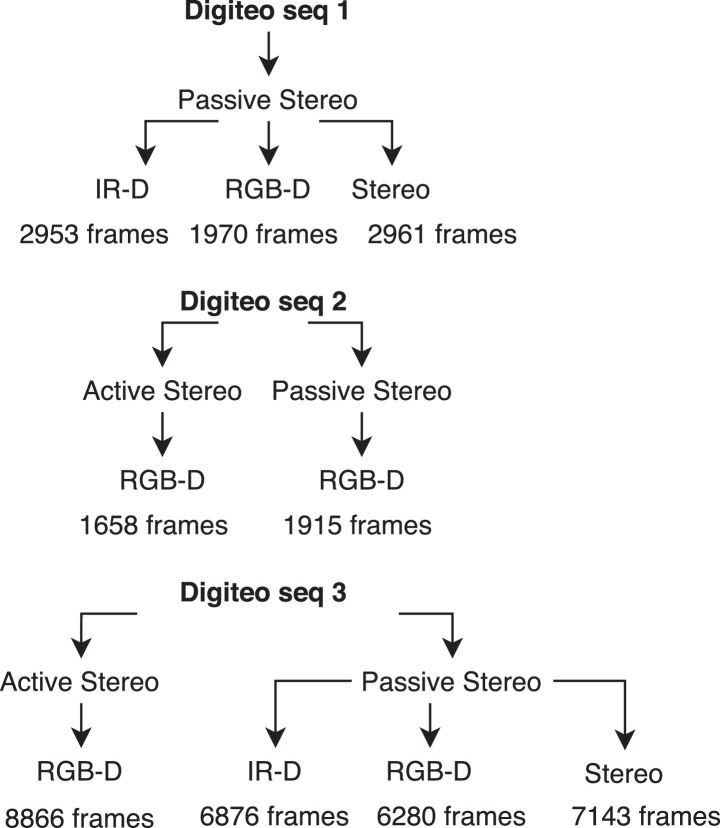
Dataset structure with the various acquisition modes.

Digiteo_seq1 and Digiteo_seq2 have varied zones in texture and brightness, also characterized by their narrow space. While for Digiteo_seq3, we have a variety in brightness and a vast space. Digiteo_seq1 consists of three acquisition modalities: Stereo, IR-D, and RGB-D in passive stereo. Digiteo_seq1 form a single loop closure over 83 m. It includes three modalities: IR-D, RGB-D, and Stereo in passive mode. Digiteo_seq2 is 58 m long with single loop closure. It has active RGB-D and passive RGB-D. The IR-D and Stereo could not be recorded because the patterns projected by the IR projector are visible, and this disturbs the detection of the primitives by the extractor. Finally, the Digiteo_seq3 recorded in the car park includes all the modalities presented before and makes the largest trajectory on 151 m with only one loop closure. The whole dataset is summarized in the Fig. 2.

**Fig. 3:**
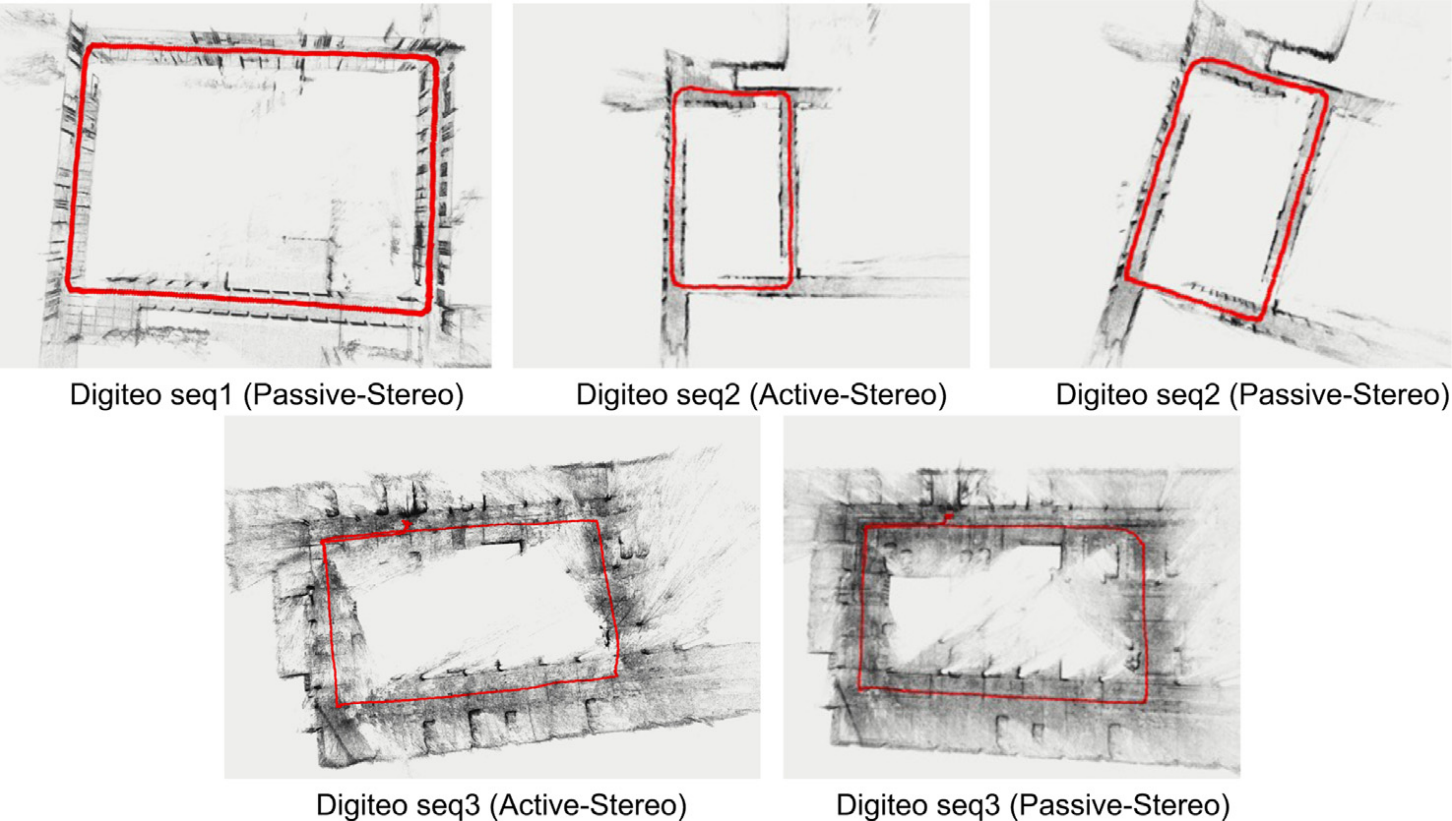
Reference trajectory in red using COLMAP.

Each dataset is provided with a file named param.yaml containing the intrinsic parameters of the camera, as well as the parameters of the ORB-SLAM2 algorithm. The dataset has a reference trajectory to make the comparison built using a Structure From Motion and Multiview-Stereo pipeline [7], [8] as shown in Fig. 3. All reference trajectories have a format compatible with the evaluation tools available on TUM RGB-D Dataset [4].

**Fig. 4:**
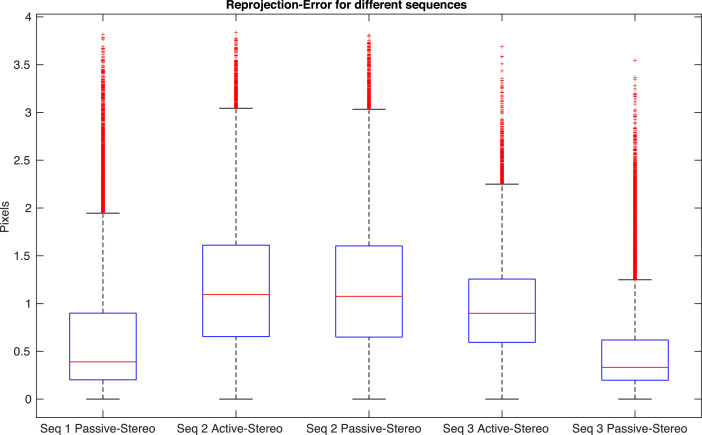
Reprojection errors of the COLMAP point cloud reconstruction.

The reprojection errors of the COLMAP point cloud reconstruction are shown in Fig. 4.

**Fig. 5:**
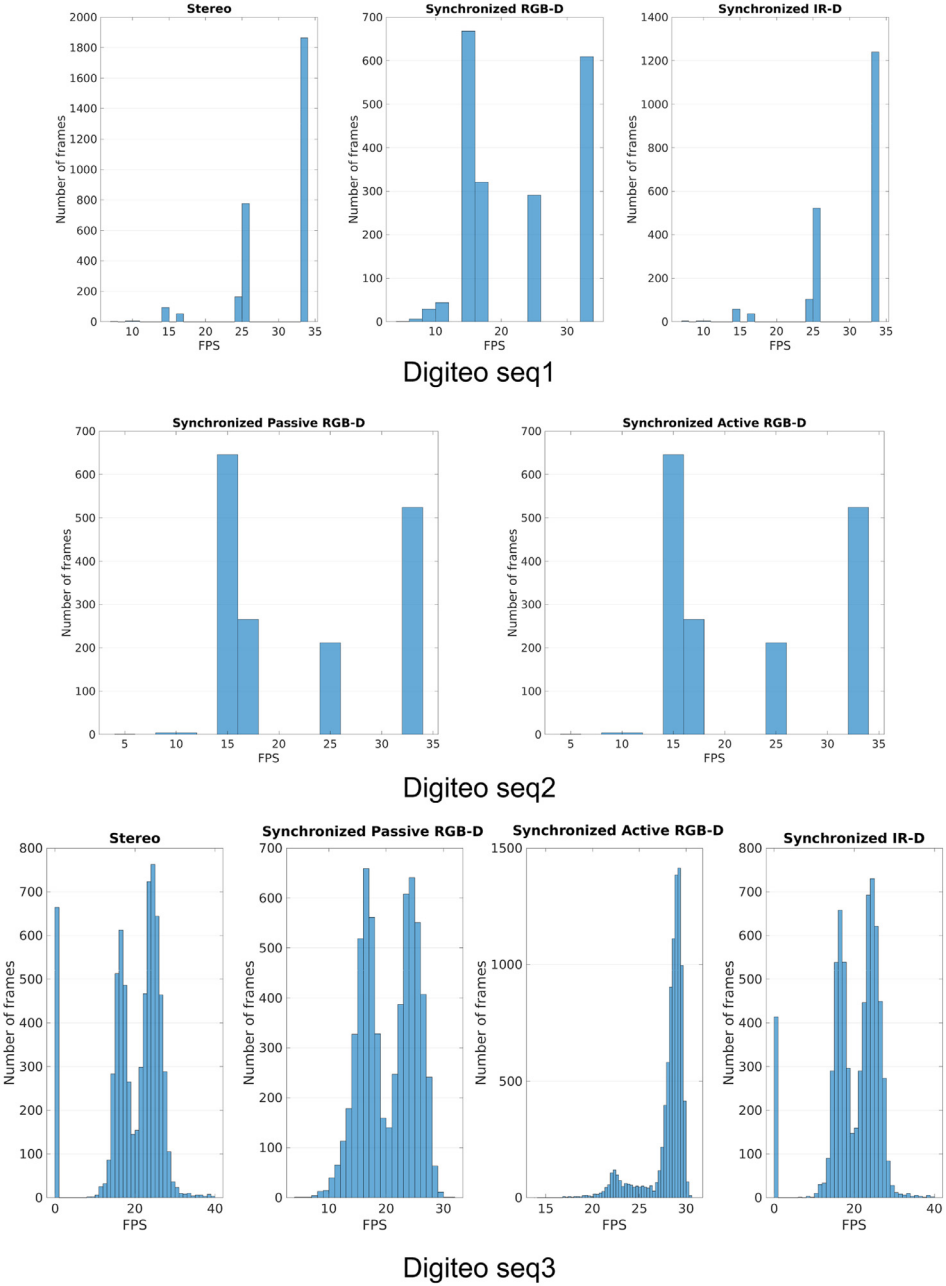
The histogram of the acquisition rate of each modality for each sequence.

**Fig. 6:**
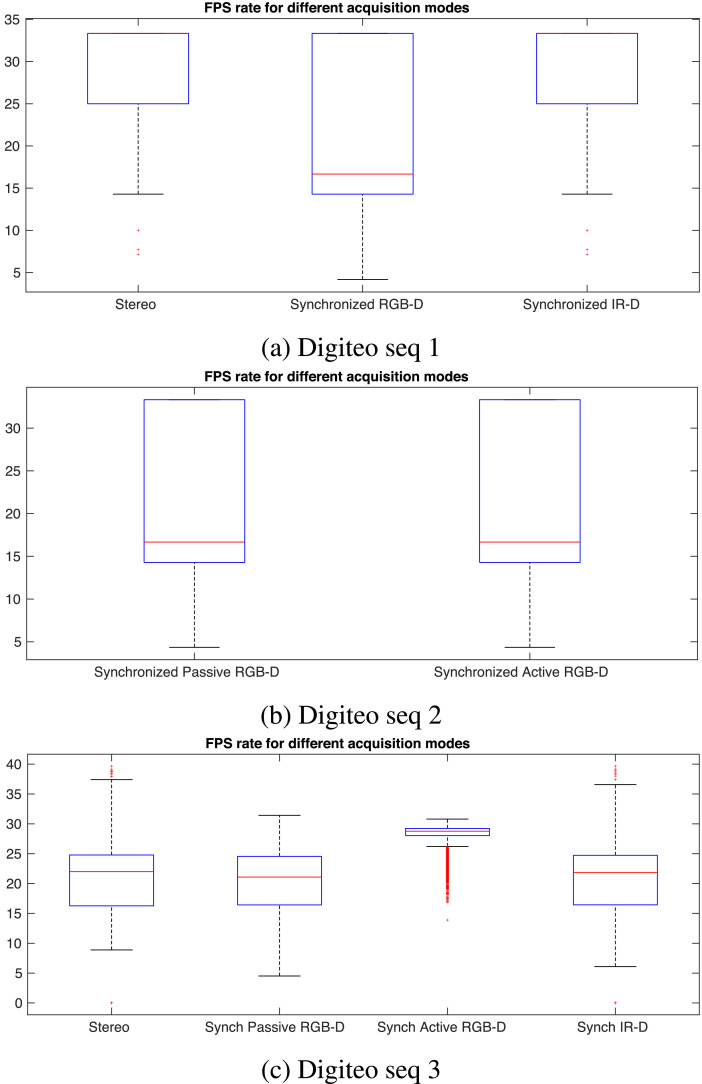
The Box plot of the acquisition rate of each modality for each sequence.

We configured the camera to acquire data at a rate of 30 FPS. The Figs. 5 and 6 represent the statistics of the acquisition rate for each sensor in the camera. The acquisition rate of RGB images with IR images ends up with a frame drop, in the case of Digiteo seq 1. Same for sequence 2. By using a laptop with more RAM, the frame rate is balanced between the three cameras. In contrast, the RGB-D active stereo is acquired alone at a rate of 30 FPS.

**Fig. 7:**
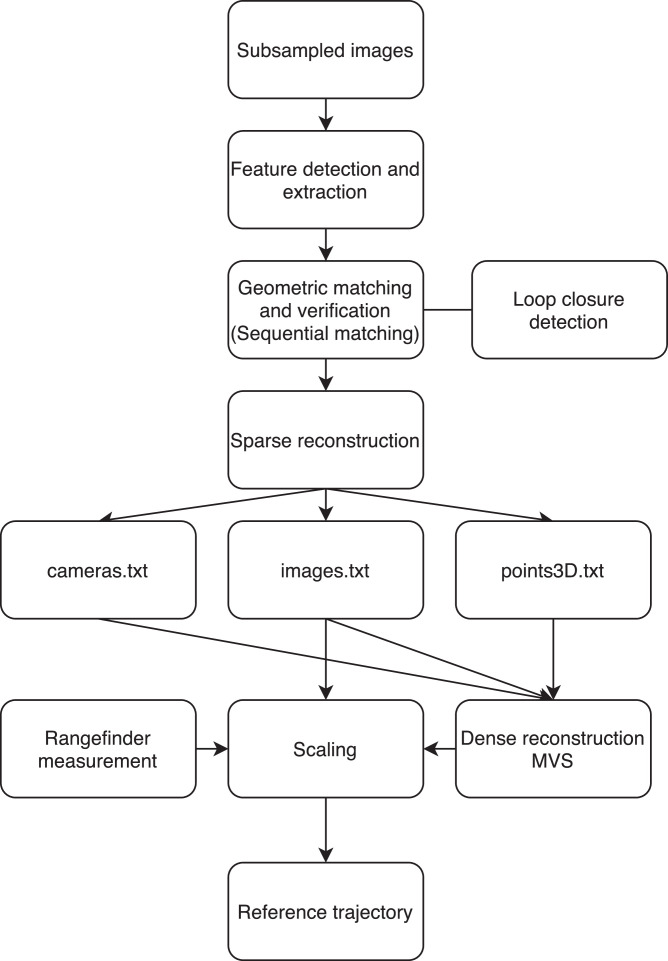
Reference trajectory process using COLMAP.

## 2 Experimental Design, Materials and Methods

The dataset was acquired using an Intel RealSense D435i camera. The camera provides stereo IR rectified, RGB-D and IR-D aligned data using realsense-ros package [9]. The images were temporally aligned after acquisition based on ROS Bag timestamps. The datasets were acquired using ROS and realsense-ros package at 30 FPS and 720p resolution without filters. A reference trajectory for comparison was created based on subsampled (subsampled by 1/5 for seq1 and seq2 and by 1/10 for seq3) monocular images of the environment using the SFM and MVS pipeline [7], [8]. The provided images are synchronized IR/RGB images with the depth images. The images used for the reference reconstruction are non-synchronized images that have been sub-sampled while keeping the timestamp of each image. Subsampled images allow for faster processing while providing sufficient visual overlap. The first step is to detect and extract features from all images and describe them using a numerical descriptor. The feature extraction uses a pinhole camera model [10] with the camera’s intrinsics and extrinsics parameters, as shown in the Tables 1 and 2. The extractor used is SIFT and executed on GPU with a maximum number of primitives of 8192.

**Table 1:** Intrinsics of the D435i camera.

	RGB	IR

Width (pixel)	1280	1280
Height (pixel)	720	720
PPX (pixel)	648.57	639.70
PPY (pixel)	363.66	356.18
Fx (pixel)	912.36	638.19
Fy (pixel)	910.26	638.19
Distortion	Inverse Brown Conrady	Brown Conrady
Coeffs	0 0 0 0 0	0 0 0 0 0
FOV (deg)	70.09 × 43.16	90.16 × 58.85

**Table 2:** Extrinsics of the D435i camera.

	“Color” to “Depth”	“Infrared 1” to “Depth”	“Infrared 2” to “Depth”
Rotation Matrix	0.9999; −0.0089; −0.0044	1; 0; 0	1; 0; 0
	0.0089; 0.9999; 0.0009	0; 1; 0	0; 1; 0
	0.0044; −0.0009; 0.9999	0; 0; 1	0; 0; 1
Translation Vector (m)	-0.0148; −0.0001; −0.0002	0; 0; 0	0.0502; 0; 0

The intrinsic parameters of the camera are set manually and shared between all images. Then, the geometric matching and verification are performed using sequential matching, which is best suited for consecutive frames with sufficient visual overlap. The overlap is set to 20, with quadratic overlap and loop detection enabled. The values of the other parameters are kept as default. Tables 3 and 4 summarize all parameters values.

**Table 3:** Feature extraction COLMAP parameters.

Camera model	pinhole
Shared for all images	Yes
Custom parameters	RGB: 912.360291, 910.268250, 648.570679, 363.666290
	IR: 638.149170, 638.149170, 639.755127, 356.510254
Max_image_size	3200
Max_num_features	8192
First_octave	−1
Num_octaves	4
Octave_resolution	3
Peak_threshold	0.00667
Edge_threshold	10
Estimate_affine_shape	No
Max_num_orientations	2
Upright	No
Domain_size_pooling	No
Dsp_min_scale	0.16667
Dsp_max_scale	3
Dsp_num_scales	10
Num_threads	−1
Use_gpu	Yes
GPU_index	−1

**Table 4:** Feature matching COLMAP parameters.

Overlap	20
Quadratic_overlap	Yes
Loop_detection	Yes
Loop_detection_period	10
Loop_detection_num_images	50
Loop_detection_num_nearest_neighbors	1
Loop_detection_num_checks	256
Loop_detection_num_images_after_verification	0
Loop_detection_max_num_features	−1
Vocab_tree_path	32K visual words (for small-scale images)
	256K visual words (for medium-scale images)
Num_threads	−1
Use_gpu	Yes
GPU_index	−1
Max_ratio	0.8
Max_distance	0.7
Cross_check	Yes
Max_num_matches	32768
Max_error	4
Confidence	0.999
Max_num_trials	10000
Min_inlier_ratio	0.250
Min_num_inliers	15
Multiple_models	No
Guided_matching	No

Loop closure detection is used through a pre-trained vocabulary tree. The GPU accelerates the matching process. Once the matching step is finished, the sparse reconstruction is launched. Data is loaded from the database into memory during this process, and the scene is expanded by incrementally registering the images from an initial image pair seed. Finally, a model can be exported, containing the camera information, the images including all the keypoints and the reconstructed pose of an image specified as the projection of the world to the camera coordinate system of an image using a quaternion and a translation vector, and finally the 3D points in the dataset. After the model is acquired, the reconstructed poses of the images are used to calculate the coordinates of the center of the projection/camera using Eq. (1).

(1) $$c_{c}=-R^{T}t$$

where $c_{c}$ is the coordinates of the camera center, $R^{T}$ is the transpose of the rotation matrix obtained from the quaternions, and t is the translation vector. For the scaling of the trajectory, we proceed to a dense reconstruction of the environment. This step consists of importing the sparse 3D model and launching the MVS, which first involves undistorting the images. The normal and depth maps are computed to be fused into a dense point cloud and finally estimating the dense surface using Poisson or Delaunay reconstruction. This dense point cloud will allow us to recover the distances of some objects with known dimensions to calculate the ratio between the distances on the point cloud and those measured with a rangefinder. This scale factor allowed us to scale our reference trajectory. Fig. 7 summarizes the process of trajectory reconstruction using COLMAP.

## Declaration of Competing Interest

The authors declare that they have no known competing financial interests or personal relationships which have or could be perceived to have influenced the work reported in this article.
